# Supplementary material for: Experience drives innovation of new migration patterns of whooping cranes in response to global change
Source: Nat Commun. 2016 Sep 6;7:12793. doi: 10.1038/ncomms12793 (PMC5025849; doi:10.1038/ncomms12793)
Supplement: Supplementary Information — Supplementary Figures 1-3 and Supplementary Tables 1-3 [file ncomms12793-s1.pdf]

Experience drives innovation of new migration patterns in response to global change: Supplementary Information

Supplementary Figure 1: Randomization test demonstrating that the effect of individual age is independent of the population aging. Each histogram represents the coefficients for age for 1000 linear mixed-effects models where age was randomized within each year. As in the main text, each model predicted site distance and included group age, grain cover, temperature change, and a spatial autocovariate as fixed effects and year as a random effect. The red line represents the coefficient associated with the actual data. In (a), groups were randomized between sites in each year but the composition of each group remained constant. The effect of age is stronger in the actual than in the randomized data (difference in values=29.82, $p<0.001$). In (b), individuals were randomized between sites in each year, so the composition of groups differed between the randomizations and the actual data, but the distribution of individual ages was the same as in the actual data. The effect of age is stronger in the actual than in the randomized data (difference in values=35.58, $p<0.001$), indicating that the effect of age in the original model is not an effect of an aging population.

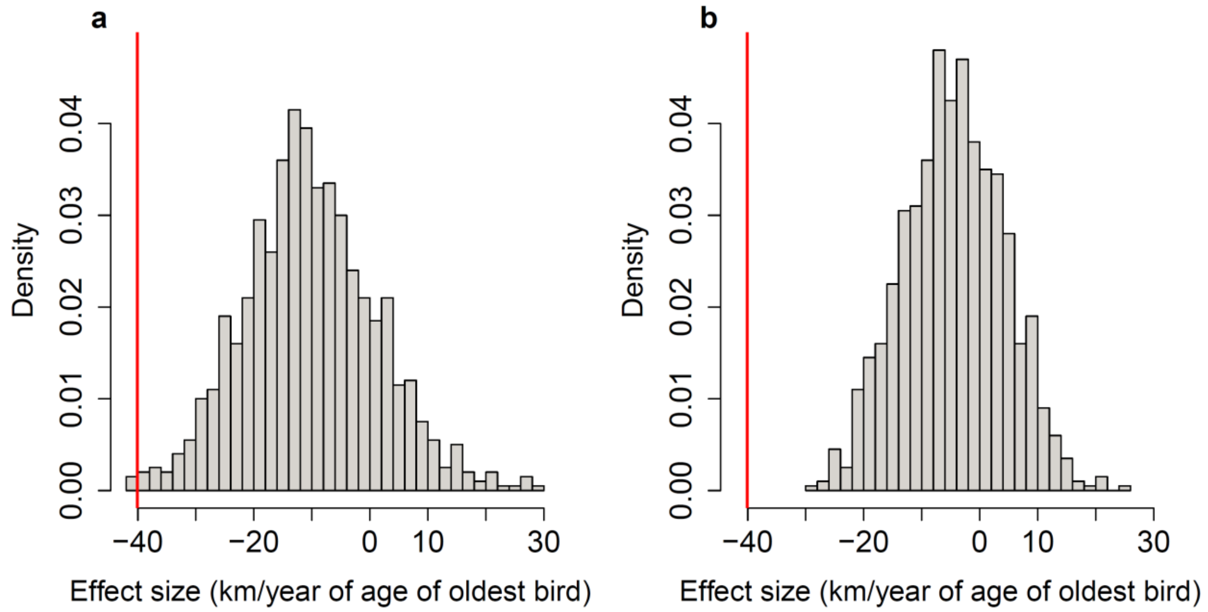

Supplementary Figure 2: Shortstopping sites. Sites depicted in teal required migration distances of less than 1200 km and were considered shortstopping sites. The asterisk shows the centroid of the population's breeding grounds (43.87°N, -89.23°E), which was used as the point of reference for calculating migration distances.

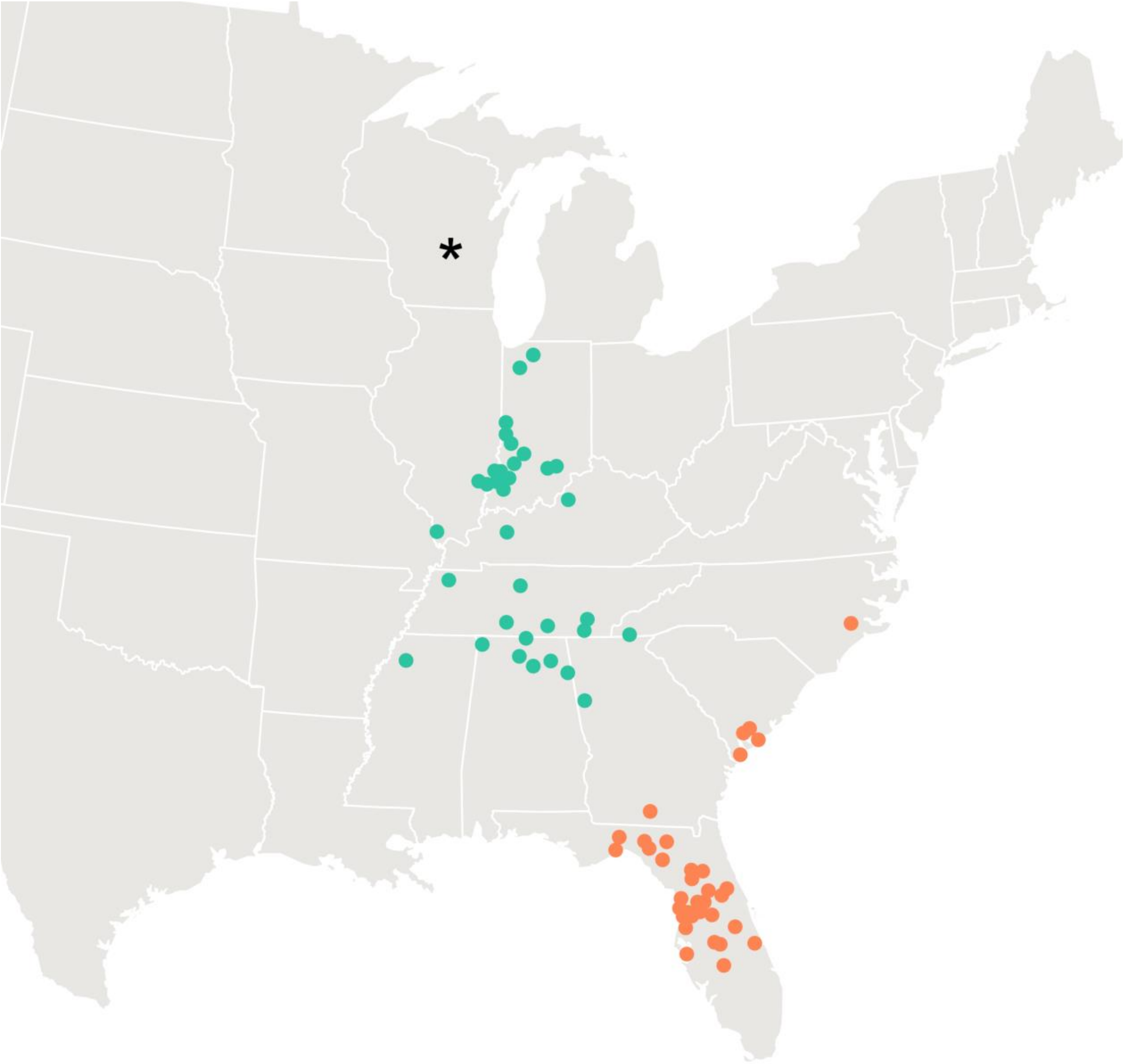

Supplementary Figure 3: Monthly distribution of whooping cranes in the eastern migratory population. Winter months when all whooping cranes are on their wintering grounds are January and February; months were any cranes are on their wintering grounds are November-April.

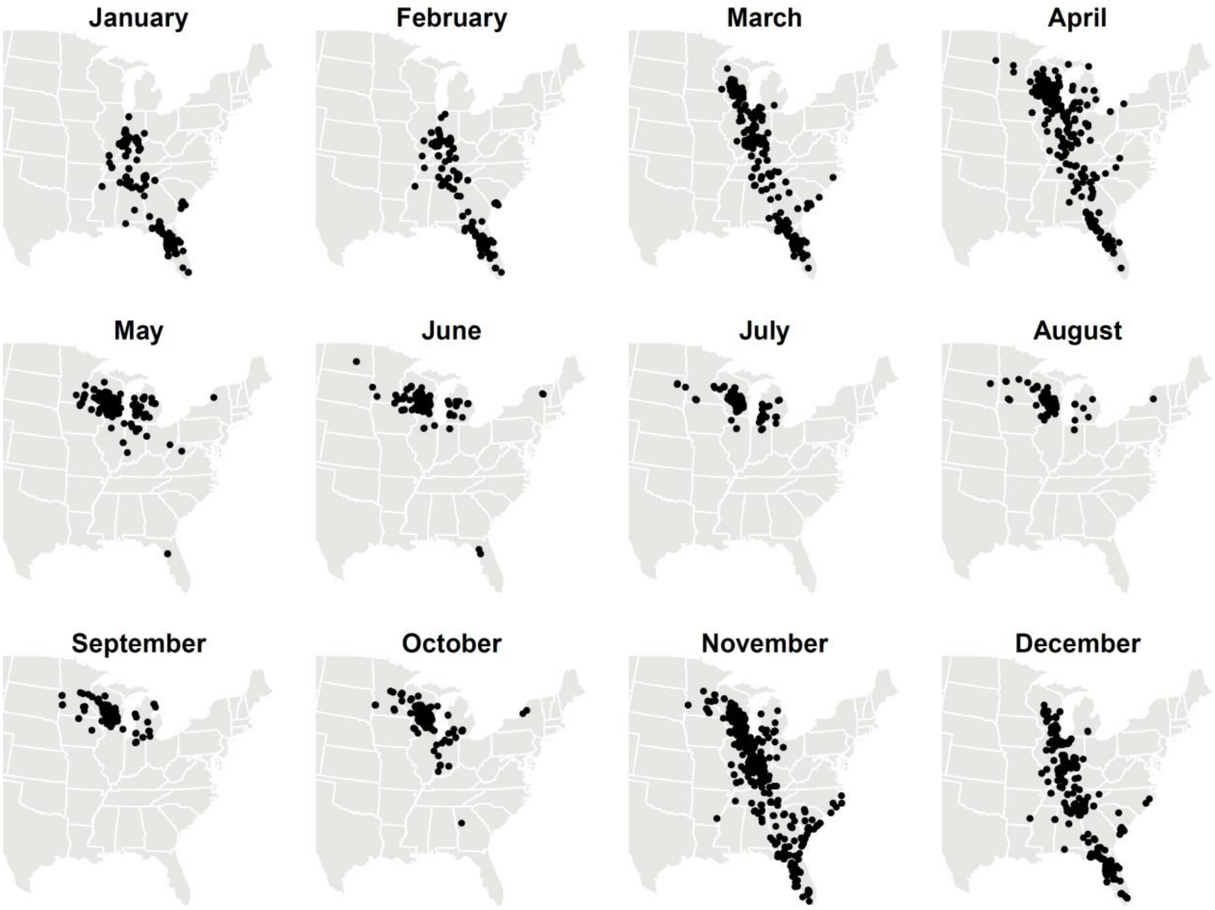

Supplementary Table 1: Model predictions using different methods of defining overwintering sites.

	**10 km location buffer**		**20 km centroid buffer**		**10 km centroid buffer**	
	Estimate	95% CI	Estimate	95% CI	Estimate	95% CI
Intercept	1661.84 ***	*1559.43, 1764.24*	1652.14 ***	*1546.31, 1757.97*	1641.54 ***	*1550.26, 1732.82*
Age of oldest bird	-40.08 **	*-66.97, -13.20*	-56.12 ***	*-84.56, -27.68*	-37.72 **	*-62.86, -12.58*
Temperature change	-257.84 ***	*-386.03, -129.64*	-258.32 ***	*-398.43, -118.20*	-232.58 ***	*-356.21, -108.95*
Grain cover	-1665.34 ***	*-2129.77, -1200.91*	-1475.36 ***	*-2024.32, -926.40*	-1685.4 ***	*-2131.96, -1238.84*
Spatial autocovariate	175.58 ***	*119.65, 231.51*	171.72 ***	*108.55, 234.89*	176.87 ***	*123.04, 230.69*
**Random effects**						
Year (s.d.)	59.44		0		44.96	
Residual	203.19		207.29		205.01	

Each model tested the distance of a site from the breeding grounds (in km) against the four explanatory variables shown. Models also included Year (the year in which a site was first used) as a random intercept. The “10 km location buffer” method is presented in the main text; in this method, two individuals are considered to be at the same overwintering site if their known locations were within 10 km of each other. The two “centroid buffer” methods considered individuals to be at the same site if the centroids of their wintering locations were within 20 and 10 km of each other, respectively. The model estimates and significance do not differ substantially between methods.

***p<0.001; **p<0.01; *p<0.05

Supplementary Table 2: Model results predicting site familiarity from individual age.

	Estimate (log km)	95% CI
Intercept	4.82***	*4.42,5.23*
Age	-0.24***	*-0.33,-0.15*
**Random Effects**		
Year (s.d)	0	
Bird ID (s.d.)	0.53	
Residual	0.94	

***p<0.001; **p<0.01; *p<0.05

This model tested log-transformed site familiarity (closest distance to site in previous years) against individual age. It also included year and individual identity as random intercepts.

Supplementary Table 3: Model results predicting probability of shortstopping from individual age and year.

	Estimate	95% CI
Intercept	-12.37***	*-16.35, -8.39*
Age	1.12***	*0.68, 1.56*
Year	0.62***	*0.2 , 0.98*

***p<0.001; **p<0.01; *p<0.05

This model tested shortstopping (a binary variable) against individual age and year. It also included individual identity as a random intercept.
